# Research on Cutting Force Modeling and Machining Performance of Discrete-Edge End Mill

**DOI:** 10.3390/mi16080923

**Published:** 2025-08-10

**Authors:** Ming Song, Minli Zheng, Siyuan Gao, Baojuan Dong, Jianping Zhu

**Affiliations:** 1 Key Laboratory of Advanced Manufacturing and Intelligent Technology, Ministry of Education, Harbin University of Science and Technology, 52 Xuefu Road, Harbin 150080, China; 43463589@163.com (M.S.); sisiaquarius@sina.com (S.G.); dongbaojuan@hibu.edu.cn (B.D.); 2 Rongcheng Campus, Harbin University of Science and Technology, 2006 College Road, Weihai 264200, China; zhujianping2017@126.com

**Keywords:** discrete-edge end mill, milling force modeling, effectiveness function of the chip separation groove, vibration suppression, surface quality

## Abstract

To address the challenges of complex cutting force formation and low prediction accuracy in discrete-edge end mills, this study proposes a precise cutting force modeling method based on an effective chip slot function. An effective chip slot function is established to quantitatively characterize the dynamic variation of cutting edge engagement along different axial positions. Based on the instantaneous uncut chip thickness theory by Altintas, a high-precision cutting force model suitable for discrete-edge tools is developed. Experimental results show that the proposed model achieves an average prediction error of 4.82%, with a maximum error below 10%, demonstrating its high accuracy and practical applicability. Comparative experiments with conventional continuous-edge end mills under identical machining conditions indicate that the discrete-edge tool can reduce cutting forces (*F_x_* by 7.2%, *F_y_* by 3.2%), significantly suppress cutting vibrations (fluctuation coefficients reduced by 13.5% and 21.9%, respectively), and lower surface roughness to approximately one-sixth of that produced by conventional tools. The results confirm that discrete-edge end mills exhibit notable advantages in machining stability, cutting force control, and surface quality, providing a solid theoretical foundation for the design and process optimization of high-performance cutting tools.

## 1. Introduction

The accelerated advancement of sectors such as aerospace, automotive manufacturing, energy equipment, and precision molds has precipitated a dramatic escalation in the demand for intricately structured components fabricated from high-performance, difficult-to-machine materials. Consequently, the domain of component manufacturing grapples with increasingly multifaceted challenges pertaining to machining requirements. Serving as the fundamental operational unit within cutting processes and an indispensable component of machine tools, the cutting tool transcends its classification as a highly consumable resource. It represents a pivotal element dictating the flexibility and efficiency of manufacturing systems. Within this context, modern manufacturing imposes progressively more stringent performance imperatives upon cutting tools, demanding not only superlative cutting capabilities and machining quality but also heightened processing efficiency and extended operational longevity. This evolving demand paradigm directly propels the trajectory of cutting tool technology toward enhanced complexity in configuration and diversity in composition [1,2,3].

The strategic modification and optimization of cutting tool geometries represent a demonstrably efficacious methodology for enhancing machining conditions, thereby facilitating the attainment of a superior cutting performance characterized by diminished vibration [4,5], prolonged tool longevity [6], elevated dimensional precision [7,8], and augmented processing efficiency [3]. End mills incorporating discrete cutting edges, distinguished by the integration of a specific number of chip grooves along their circumferential flutes, offer distinct advantages including superior chip evacuation efficacy, reduced milling forces, and extended operational service life [9]. In recent years, this category of cutting tools has garnered significant research attention from scholars globally. Investigations by Gerami et al. [10] substantiate that chip grooves effectively partition continuous chips into narrower and thinner segments, resulting in a pronounced reduction—particularly in radial components—in cutting forces, concurrently optimizing the distribution of cutting loads. Research by Samsudeensadham et al. [11] provides empirical evidence that chip grooves significantly enhance the chip-breaking capability of the tool. Collectively, these findings underscore that the structural design of chip grooves on the side flanks of end mills contributes substantially to dynamic chip segmentation and the amelioration of cutting load distributions. Meanwhile, some scholars have conducted in-depth research on the cutting mechanism of discrete-edge tools. Wang et al. [12] examined the influence of chip-breaking parameters on the cutting process, while Fang et al. [13] developed predictive models for chip formation specific to discrete-edge tools. Jawahir et al. [14] conducted an analysis of wear behavior in such tools, and Ni et al. [15] investigated predictive models for the cutting performance of broaching tools. Crucially, the precise prediction of cutting forces constitutes a critical foundation for optimizing process parameters, mitigating machining vibrations, and extending the functional lifespan of cutting tools.

End mills featuring discrete cutting edges with integrated chip grooves demonstrably enhance chip evacuation efficacy and augment processing efficiency. However, a comprehensive elucidation of their underlying cutting mechanisms remains elusive. Contemporary milling force models are conventionally categorized, based on their methodological foundations, into four distinct paradigms: empirical models [16,17], analytical models [18,19,20,21], finite element models [22,23], and artificial intelligence-based models [24,25]. Notably, the extant literature reveals a conspicuous absence of dedicated research focused on cutting force modeling specifically for discrete-edge cutting tools. Consequently, the establishment of a high-fidelity cutting force model for discrete-edge end mills, coupled with an in-depth investigation into their machining performance, possesses substantial theoretical significance and considerable engineering merit.

This research addresses the critical challenges associated with modeling the mechanical behavior and refining the mechanistic understanding of discrete-edge end mills during the cutting process. By establishing a chip groove effectiveness function, a high-precision predictive model for milling forces is developed. Through systematic theoretical modeling and rigorous experimental validation, the study elucidates the inherent vibration-damping mechanism facilitated by the discrete-edge structure and quantifies its concomitant contribution to enhanced machining quality. The resultant findings not only furnish a novel theoretical framework for advancing the study of cutting mechanisms in discrete-edge end mills but also provide a robust foundation for optimizing process parameters in practical engineering applications. This work, therefore, holds cardinal significance for propelling the development of next-generation, high-performance cutting tools.

## 2. Modeling of Milling Force for Discrete-Edge End Mills

**Figure 1:**
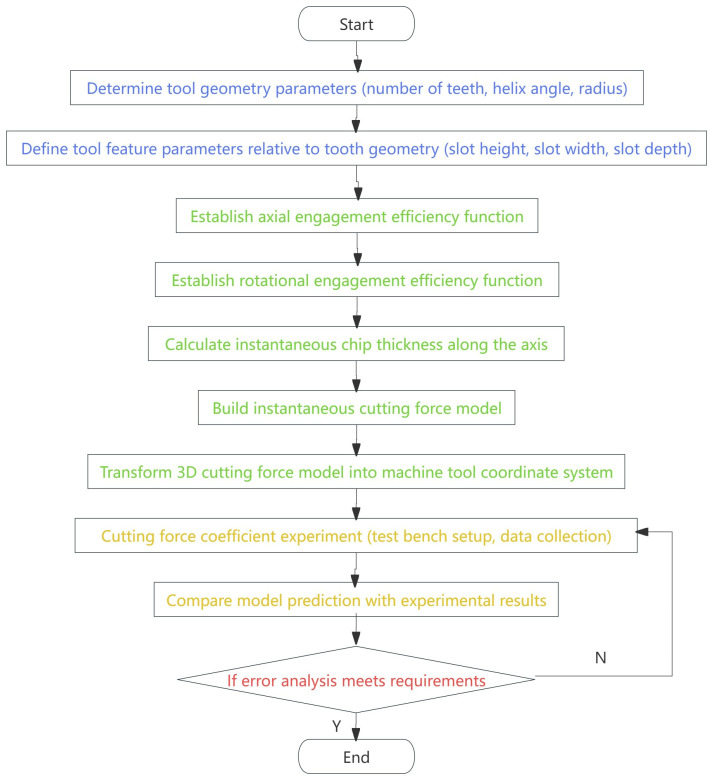
Flowchart of milling force modeling.

The strategically designed chip separation groove on the side cutting face of the end mill facilitates dynamic chip separation and optimizes cutting loads. This mechanism relies on the periodic opening and closing of the chip chute combined with the dynamic entry of the cutting edge, which induces periodic discontinuity in the cutting process. This phenomenon improves cutting force distribution, reduces vibration levels, and enhances the tool’s load control capability. Following Altintas’ theory, milling force modeling employs a hierarchical progressive approach. The mathematical model of milling force is developed by integrating the tool–workpiece kinematic relationship and incorporating key parameters, including the chip slot effectiveness function, rotational effectiveness function, and the number of effective cutting edges. The modeling process of the milling force of the discrete-edge end mill is shown in Figure 1.

### 2.1. Tool Geometry Parameters

The critical geometric parameters of an end mill comprise the number of teeth (*N*), the helix angle (*β*), and the tool radius (*R*). The helix angle induces a phase lag effect along the cutting edge, where each point lags behind the tool’s endpoint. This lag effect varies with the axial position. At a given axial height (*z*), the lag angle is defined by the following equation:

(1) $$\psi =\frac{z\cdot \tan\beta }{R}.$$

In a symmetrical discrete-edge end mill, the cutting edges are uniformly distributed about the circumference. The inter-tooth angle is given by the following:

(2) $$\phi_{p}=\frac{2\pi }{N}.$$

Assuming zero initial angular displacement (*ϕ*_10_ *=* 0) for the first cutting edge’s endpoint, the initial angular displacement of the i-th cutting edge at axial height *z* is given by the following:

(3) $$\phi_{i}\left(z\right)=(i-1)\phi_{p}+\frac{z\cdot \tan\beta }{R}.$$

**Figure 2:**
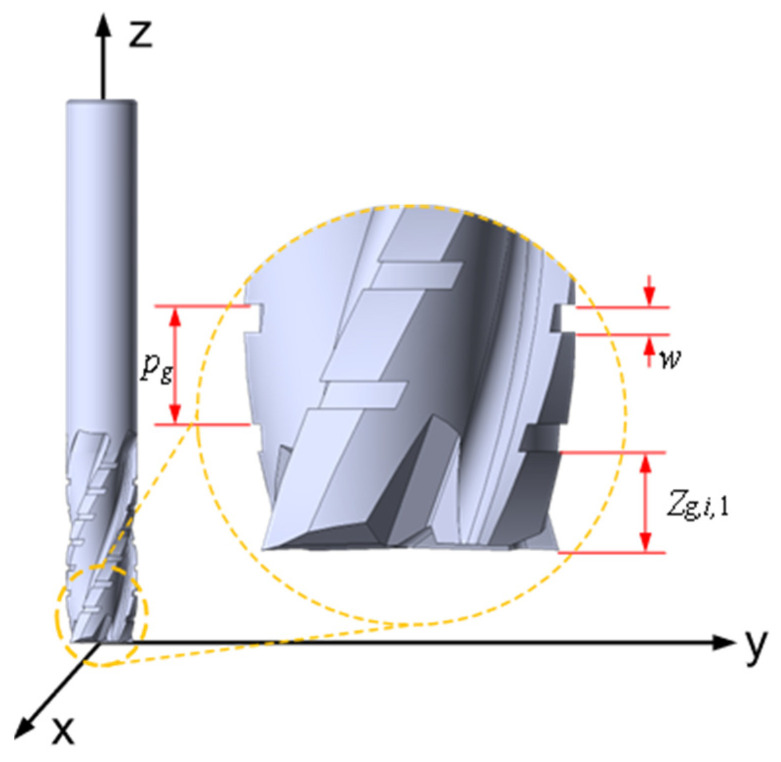
Discrete parameter schematic diagram of discrete-edge end mill.

The discrete-edge end mill’s geometric configuration is characterized by four fundamental parameters: the axial chip flute width (*ω_g_*), the axial chip flute pitch (*p_g_*), the axial reference position of the first flute (*z*_*g*,*i*,1_), and the axial discrete interval ratio (*η* = *ω_g_*/*p_g_*). These parameters collectively define the tool’s cutting geometry, with their spatial relationship visually presented in Figure 2.

To precisely characterize the axial positioning of each cutting edge, the reference position for the initial chip groove of odd-numbered cutting edges is designated as *z_g_*_,1,1_ = *z*_0_. Consequently, the corresponding reference position for the initial chip groove of even-numbered cutting edges is systematically defined as follows:

(4) $$z_{g,2,1}=z_{0}+\frac{p_{g}}{2}.$$

Consequently, the axial reference position of the primary chip-breaking groove for the *i*-th cutting edge can be universally formulated as follows:

(5) $$z_{g,i,\;\;\;1}=z_{0}+\frac{p_{g}}{2}\cdot \left[1-\mathrm{mod}(i,2)\right].$$

The mathematical expression for the axial reference height *z*_*g*,*i*,*j*_ of the j-th chip groove corresponding to the *i*-th cutting edge is as follows:

(6) $$z_{g,i,j}=z_{0}+\frac{p_{g}}{2}\cdot \left[1-\mathrm{mod}(i,2)\right]+(j-1)p_{g}.$$

The axial extent of the *j*-th chip-breaking groove on the *i*-th cutting edge is mathematically described by the following:

(7) $$z\in \left[z_{g,i,j},z_{g,i,j}+\omega_{g}\right].$$

The mathematical expression for the distribution area of each chip groove of the *i*-th cutting edge along the axial direction can be expressed as follows:

(8) $$z\in \sum_{j=1}^{N_{g,i}}\left[H(z-z_{g,i,j})-H(z-z_{g,i,j}-\omega_{g})\right].$$

In the formulation, the unit step function *H*(·) is defined as follows:

(9) $$H\left(x\right)=\left\{\begin{array}{cc} 1, & x\geq 0,\\ 0, & x<0. \end{array}\right.$$

In the formulation, *N*_*g*,*i*_ denotes the number of chip-breaking grooves on the *i*-th cutting edge, determined by the following:

(10) $$N_{g,i}=\left\lceil \frac{a_{p}-z_{g,i,\;\;\;1}}{p_{g}}\right\rceil .$$

In the formula, $\left\lceil x\right\rceil$ represents the rounding operation downwards—that is, when $a_{p}\leq z_{g,i,1}$ is $N_{g,i}=0$, when $z_{g,i,1}\leq a_{p}\leq z_{g,i,1}+p_{g}$ is $N_{g,i}=1$, etc.

### 2.2. Chip-Breaking Groove Effectiveness Function and Rotational Effectiveness Function

Due to the introduction of the chip-breaking groove, the effective number of cutting edges *Z_eff_*(*z*) of the discrete-edge end mill at different axial heights will change with the variation of the axial position.

To quantitatively describe this change, the concept of the chip-breaking groove effectiveness function $\gamma_{slot,i}(z)$ is proposed in this study. When the *i*-th cutting edge is within the chip-breaking groove range at height *z*, the function value is set to 1 (indicating that this cutting edge does not participate in the cutting process); otherwise, the function value is 0. The definition of the function is as follows:

(11) $$\gamma_{slot,i}\left(z\right)=\sum_{j=1}^{N_{i}}\left[H\left(z-z_{g,i,j}\right)-H\left(z-z_{g,i,j}-\omega_{g}\right)\right].$$

At a specific height *z*, the actual number of cutting edges involved in the cutting process can be obtained by subtracting the number of cutting edges located in the chip-breaking groove from the total number of cutting edges *N*. The specific expression is as follows:

(12) $$Z_{eff}(z)=N-\sum_{i=1}^{N}\gamma_{slot,i}(z).$$

**Figure 3:**
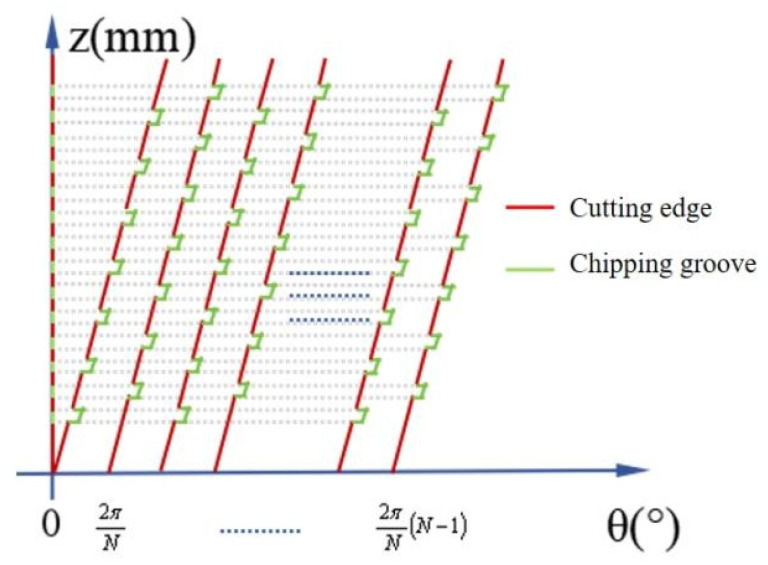
Schematic diagram of the extension of the cutting edge of a discrete-edge end mill tool.

As shown in Figure 3, the total of all the red cutting edges at a certain height represents the actual number of effective cutting edges, denoted as $Z_{eff}(z)$.

Set the rotational speed of the tool to *n*. The formula for calculating the rotational angle within time t can be expressed as follows:

(13) $$\theta =\frac{2\pi \cdot n}{60}\cdot t.$$

At this point, the position angle $\theta_{i}(z,\theta )$ of the *i*-th cutting edge at height *z* can be expressed as follows:

(14) $$\theta_{i}(z,\theta )=\theta -\phi_{i}\left(z\right).$$

The necessary condition for achieving an effective cutting state at any position on the cutting edge of a tool is that the angle at that position lies within the effective cutting range formed by the entry angle and the exit angle. Therefore, in order to ensure that the angle $\theta_{i}(z,\theta )$ of the periodic rotational motion always falls within the standard range $\left[0,2\pi \right)$, it is necessary to perform periodic processing on it to obtain the periodic angle ${\tilde{\theta }}_{i}(z,\theta )$ of the cutting edge. Its mathematical expression can be described as follows:

(15) $${\tilde{\theta }}_{i}(z,\theta )=\mathrm{mod}\left[\theta_{i}(z,\theta ),2\pi \right].$$

Based on this, the rotational validity function $\gamma_{i}(\theta ,z)$ can be further defined:

(16) $$\gamma_{i}\left(\theta ,z\right)=H\left[{\tilde{\theta }}_{i}\left(z,\theta \right)-\phi_{st}\right]-H\left[{\tilde{\theta }}_{i}\left(z,\theta \right)-\phi_{ex}\right].$$

In Equation (16), the calculation formulas for the cutting angle $\phi_{st}$ and the cutting angle $\phi_{ex}$ are determined respectively based on the milling method (either normal milling or reverse milling).

For sequential milling:

(17) $$\left\{\begin{array}{l} \phi_{st}=\pi -\arccos\left(1-\frac{a_{e}}{R}\right),\\ \phi_{ex}=\pi . \end{array}\right.$$

For reverse milling:

(18) $$\left\{\begin{array}{l} \phi_{st}=0,\\ \phi_{ex}=\pi -\arccos\left(1-\frac{a_{e}}{R}\right). \end{array}\right.$$

This takes into account the influence of the rotational state of the cutting tool and the axial chip-breaking grooves on the cutting process. When the rotational effectiveness function is satisfied and the effectiveness function of the chip-breaking grooves is not satisfied, the cutting edge can truly exert its cutting effect only at a specific position and angle. Therefore, the comprehensive effectiveness function is defined as follows:

(19) $$\gamma_{eff,i}(\theta ,z)=\gamma_{rot,i}(\theta ,z)\cdot [1-\gamma_{slot,i}(z)].$$

### 2.3. Cutting Force Model

Based on the theoretical model of the cutting edge efficiency function, it is possible to further clarify the instantaneous cutting thickness of each cutting edge at any height *z* and position angle θ. The mathematical expression is as follows:

(20) $$h_{i}(\theta ,z)=\gamma_{eff,i}(\theta ,z)\cdot f_{z}(z)\cdot \sin({\tilde{\theta }}_{i}(z,\theta )).$$

Here, $f_{z}\left(z\right)$ represents the feed per tooth, and its calculation formula is as follows:

(21) $$f_{z}(z)=\frac{f_{r}}{Z_{eff}(z)}.$$

In the formula, $f_{r}$ represents the feed rate per unit revolution.

**Figure 4:**
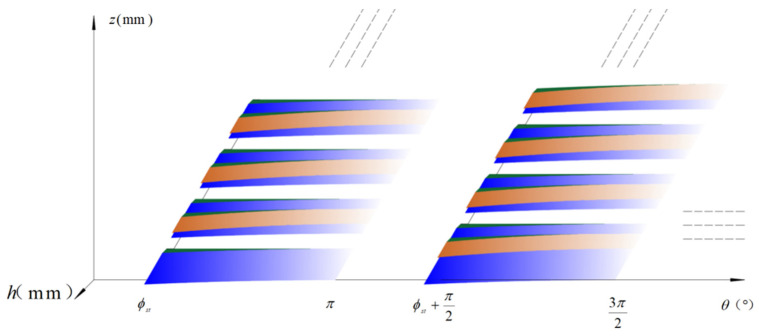
Schematic diagram of the distribution of chip-cutting grooves on the cutting edge of a discrete-edge end mill and the variation in cutting thickness.

Figure 4 shows the distribution of the chip channels on the cutting edge and the dynamic changes in the cutting thickness during the simultaneous milling of two teeth of a four-tooth discrete-edge end mill. The diverse colors in the figure depict the characteristics of the cutting thickness variation of the cutting edge during the cutting process, which is attributed to the layout of the chip breaker. The white region designates the location of the chip breaker of the current cutting edge. The blue region represents the cutting thickness distribution at this axial height when none of the cutting edges has a chip breaker. The orange region reflects the alteration in the cutting thickness of the current cutting edge when influenced by the chip breaker of the preceding edge. The green region demonstrates the difference in the cutting thickness along the radial direction of the same cutting edge at a specific axial height, resulting from the spatial distribution of the chip breaker. The gradation of all colors corresponds to the trend of the cutting thickness changing from larger to smaller values.

Based on the theory of elemental cutting force, the coefficients of tangential, radial, and axial shear forces per unit length, as well as the ploughing force coefficient, are respectively defined as $K_{tc}$, $K_{rc}$, $K_{ac}$, $K_{te}$, $K_{re}$, and $K_{ae}$. Therefore, the elemental cutting forces along the tangential, radial, and axial directions of the tool coordinate system can be expressed as follows:

(22) $$dF_{t,i}=\gamma_{eff,i}(\theta ,z)\cdot \left(K_{tc}\cdot h_{i}(z,\theta )+K_{te}\right)\cdot dz,\;dF_{r,i}=\gamma_{eff,i}(\theta ,z)\cdot \left(K_{rc}\cdot h_{i}(z,\theta )+K_{re}\right)\cdot dz,\;dF_{a,i}=\gamma_{eff,i}(\theta ,z)\cdot \left(K_{ac}\cdot h_{i}(z,\theta )+K_{ae}\right)\cdot dz.$$

Regarding the spatial position conversion relationship between the tool coordinate system and the machine tool coordinate system, the component forces of the micro-cutting force in the tangential, radial, and axial directions are converted to the *X*-, *Y*-, and *Z*-axes of the machine tool coordinate system, and the conversion relationship is expressed as follows:

(23) $$\left[\begin{array}{c} dF_{x,i}\\ dF_{y,i}\\ dF_{z,i} \end{array}\right]=\left[\begin{array}{ccc} \cos({\tilde{\theta }}_{i}(z,\theta )) & -\sin({\tilde{\theta }}_{i}(z,\theta )) & 0\\ \sin({\tilde{\theta }}_{i}(z,\theta )) & \cos({\tilde{\theta }}_{i}(z,\theta )) & 0\\ 0 & 0 & 1 \end{array}\right]\left[\begin{array}{c} dF_{t,i}\\ dF_{r,i}\\ dF_{a,i} \end{array}\right].$$

During the process of constructing the three-dimensional cutting force model in the machine tool coordinate system, the cutting force component expressions of each cutting edge in the machine tool coordinate system can be obtained through the integration method. By considering the combined effect of all effective cutting edges, the overall cutting force of the tool in the machine tool coordinate system can be expressed as follows:

(24) $$\left\{\begin{array}{c} F_{x}=\int_{0}^{a_{p}}\sum_{i}^{N}dF_{x,i},\\ F_{y}=\int_{0}^{a_{p}}\sum_{i}^{N}dF_{y,i}\\ F_{z}=\int_{0}^{a_{p}}\sum_{i}^{N}dF_{z,i}. \end{array}\right.,$$

## 3. Experimental Verification of the Cutting Force Model for Discrete-Edge End Mills

### 3.1. Identification of Cutting Force Coefficients

**Figure 5:**
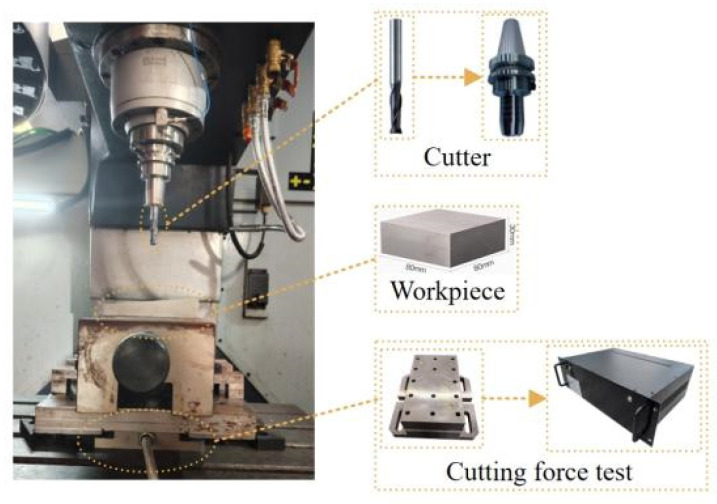
Parameter identification experimental platform.

To accurately obtain the key coefficients of the cutting force model in Equation (22), a systematic measurement experiment was designed and conducted on the side milling cutting force. The experiment was carried out on an HDCNC VC1056 five-axis machining center, and the workpiece material was 45 steel. The cutting force signals were dynamically collected using a high-precision piezoelectric three-directional force sensor (model YD15-III1710), with a sampling frequency of 10 kHz to ensure high time resolution and measurement accuracy of the cutting force signals. The workpiece was rigidly clamped using a specially designed fixture to ensure the rigidity of the clamping and the stability of the experimental process. The layout of the experimental platform is shown in Figure 5.

The experiment adopted the side milling condition [26]. The side milling condition was selected to ensure that the chip-breaking groove structure could fully participate in the cutting process, avoiding the processing vibration and instability caused by the cutting width being equal to the tool diameter (12 mm) during slot milling. This scheme has been verified through preliminary experiments, and the process parameters were designed based on the relevant literature to ensure that the experimental design is reasonable and the data are stable and reliable.

After the collected cutting force signal was preprocessed, the data during the stable cutting stage were selected for the identification of the cutting force coefficient. Based on the difference square sum of the theoretical cutting force and the measured force in Formula (22), a target function was constructed, and the nonlinear least squares fitting algorithm (lsqcurvefit function) in MATLAB R2021a was used to iteratively solve it. The initial cutting force coefficient was estimated from the literature and preliminary experimental results to ensure the convergence and stability of the fitting process.

**Figure 6:**
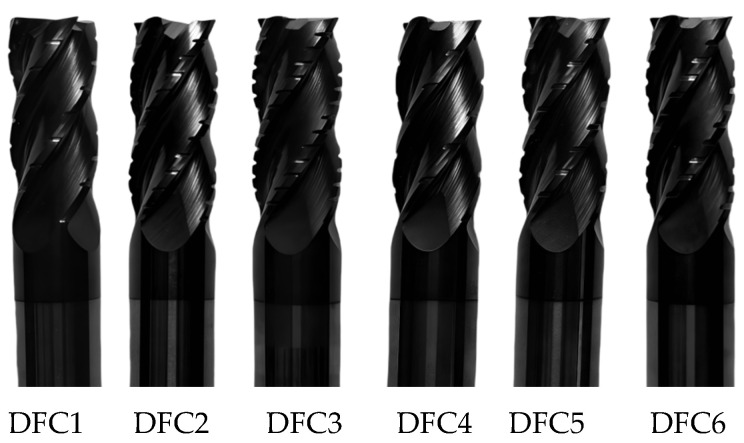
Experimental discrete-edge end mill.

The cutting tool was a four-edge discrete-edge end mill with a customized edge grinding process, as shown in Figure 6. The diameter of the tool was Φ12 mm and the helix angle was 30°. The discrete parameters are detailed in Table 1.

To determine the cutting force coefficient, detailed cutting process parameters are shown in Table 2.

When an end mill is used for side milling, the components *F_x_* and *F_y_* of the tangential force are the main parameters for tool design and process optimization. They have a decisive influence on the force stability of the tool, the processing quality of the workpiece surface, and the machining accuracy. In contrast, the axial cutting force in side milling operations is usually relatively small, and its impact on the stiffness of the process system and the processing quality is relatively secondary. Based on this, this study focuses on conducting in-depth analysis and model verification of *F_x_* and *F_y_* that play a dominant role in the side milling process, without further exploring the axial force *F_z_*, which is secondary.

To accommodate the nonlinear characteristics of the model, the nonlinear least squares method was adopted to identify the cutting parameters. Through calculation, the specific values of each cutting force coefficient were obtained: $K_{tc}=11765.886$, $K_{rc}=6113.672$, $K_{te}=-36.563$, $K_{re}=-42.025$.

### 3.2. Verification of the Cutting Force Model

Based on the milling force coefficient obtained in Section 3.1, combined with theoretical derivation and experimental data, the detailed results are presented in Table 3.

The milling process is a typical intermittent cutting process. The cutting force fluctuates periodically with the rotation of the tool, and the instantaneous cutting force exhibits significant dynamic characteristics. To accurately reflect the stable cutting state during the processing, the experimental cutting force shown in Table 3 in this study adopts the time average value within the stable cutting stage as a representative indicator. Specifically, the original cutting force signal is preprocessed, the unstable data during the start and stop stages are eliminated, and the force signal during the stable working condition period is selected. Subsequently, the arithmetic average of each force component within this period is calculated as the representative value of the experimental measurement of the cutting force. The theoretical cutting force is based on the constructed instantaneous cutting force model. By integrating the cutting force responses of all effective cutting edges within the selected time interval, the corresponding time-averaged predicted value is obtained.

**Figure 7:**
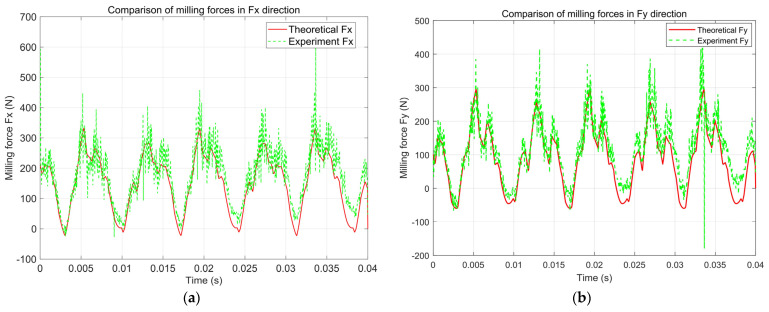
Theoretical and experimental prediction comparison of side milling cutting force signals for discrete-edge milling tools. (**a**) Comparison of milling forces in the *F_x_* direction; (**b**) Comparison of milling forces in the *F_y_* direction.

**Figure 8:**
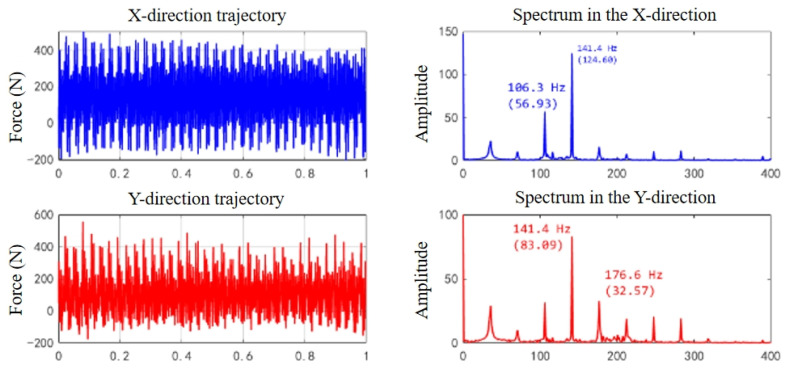
Experimental measurement of cutting force during the side milling process of discrete-edge end mill in time domain and frequency spectrum.

**Figure 9:**
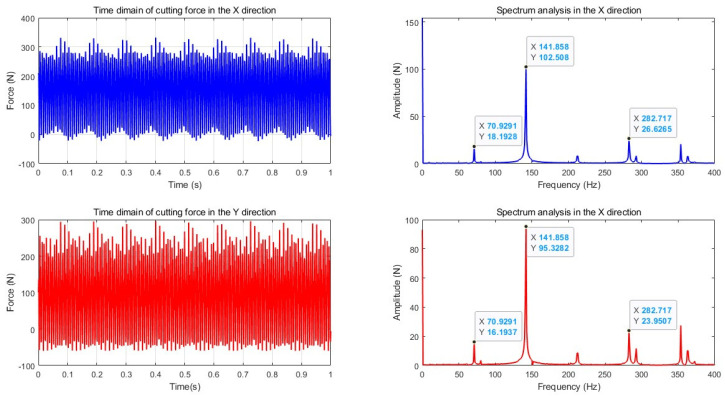
Theoretical prediction of the time domain and frequency domain cutting forces of side milling with discrete-edge end mills.

The data analysis in Table 3 shows that in the *F_x_* direction, the error range is between 1.01% and 7.05%, with the fifth group of data having the largest error (experimental value of 142.4 N, theoretical value of 153.2 N), and the average error is 4.82%; in the Fy direction, the error range is between 2.98% and 7.44%, and the fifth group of data also shows the largest error (experimental value of 109.37 N, theoretical value of 101.48 N), with an average error of 4.32%. The average errors in both the *F_x_* and *F_y_* directions are limited within ±5%, and the maximum error is controlled within ±10%, thereby verifying the high prediction accuracy and good engineering applicability of the constructed cutting force model under the side milling condition. The research results show that this model can accurately capture the distribution characteristics of cutting force under the discrete chip structure and exhibits good stability and reliability. To verify the accuracy of the constructed milling force theoretical model in predicting the milling force of the discrete-edge face end mill under actual processing conditions, an in-depth comparative analysis in the time domain and frequency domain was conducted. The theoretical and experimental prediction comparison results of the *F_x_* and *F_y_* cutting force signals of the DFC1 tool are shown in Figure 7, Figure 8 and Figure 9.

Figure 7 shows the comparison of the cutting force signals in the *X* (*F_x_*) and *Y* (*F_y_*) directions during the side milling process of the proposed discrete-edge end mill. The heights and fluctuation trends of the curves are highly consistent, indicating that the model can accurately reflect the periodic variation characteristics and amplitude of the cutting force. This comparison verifies the effectiveness and accuracy of the cutting force model in the time domain.

A comprehensive comparative analysis was conducted based on the average cutting force, dominant frequency position, and amplitude of the signals, with the results summarized in Table 4.

(1) Time domain signal comparison

The overall fluctuation trends of the cutting force signals from theory and experiment are consistent, with both showing typical periodic fluctuation characteristics. The average predicted cutting force values in the *X*, *Y*, and *Z* directions by theory are close to the experimental measurement values, with the error controlled within 10%, indicating that the model can accurately describe the static force level during the side milling process of the discrete-edge end mill.

(2) Spectrum signal comparison

The theoretical and experimental main frequency positions are highly consistent. The three main frequencies are approximately 141.6 ± 0.3 Hz, indicating that the model accurately reflects the discrete tooth periodic cutting excitation characteristics.

The theoretical and experimental main frequency amplitudes are relatively close. The main frequency amplitudes in the *X* and *Y* directions have errors of about 20%, while in the *Z* direction, due to the influence of factors such as clamping stiffness and sensor sensitivity, the error is relatively large, but the overall trend still basically matches.

In terms of secondary frequency components, both the theoretical and experimental results showed frequency components such as 106.3 Hz and 176.6 Hz, indicating that the model can reflect the multi-tooth interference effect and the superposition effect of the feed excitation frequency.

From the above comparative analysis, it can be seen that the prediction error of the static component (average force) is less than 10%, the error of the main frequency position is less than 0.5%, the error of the main frequency amplitude in the *X* and *Y* directions is less than 20%, the secondary frequencies are completely consistent with the prediction, and the trend of the time domain waveform is consistent. Based on the above data, it can be concluded that the overall prediction accuracy of the model is high, effectively reflecting the true milling force characteristics during the side milling process of the discrete-edge end mill and proving the validity and accuracy of the model.

## 4. Performance Test of Discrete-Edge End Mill

### 4.1. Experiment Conditions

To investigate the cutting performance of the discrete-edge end mill, a cutting performance comparison experiment of the discrete-edge end mill (DFC1) and a conventional end mill (continuous-edge type, as a control) was conducted based on the original equipment. The Surface morphology features were measured using a confocal laser microscope (OLS5000, Olympus, Tokyo, Japan). The geometric parameters and cutting process parameters of the two types of tools were the same as shown in Table 5.

### 4.2. Analysis of Cutting Force Comparison

The cutting forces of the two types of tools were compared and analyzed from two dimensions: time domain characteristics and spectral characteristics. The stable cutting segment within the middle 2 s of the experimental data was selected for analysis. Table 6 summarizes the statistical characteristics of the cutting forces of the conventional end mill and the discrete-edge end mill, including the three-directional average force, fluctuation coefficient, main frequency, and main frequency amplitude.

From the results of the comparison table of cutting force statistical characteristics, the following can be concluded:

(1) In the *X* and *Y* directions, the average force of the discrete-edge face end mill was 86.18 N and 53.74 N, respectively. Compared with the force of 92.85 N and 55.53 N of the conventional face end mill, it achieved a reduction of 7.2% and 3.2%, respectively.
(2) The fluctuation coefficients of the discrete-edge end mill in the *X* and *Y* directions were 0.64 and 0.57, respectively. Compared with the coefficients of 0.74 and 0.73 of the conventional end mill, they achieved a reduction of 13.5% and 21.9%, respectively.
(3) The main frequencies of the two cutting tools in the *X*, *Y*, and *Z* directions were all 71 Hz, exactly the same, and they both belong to the side-edge cutting spectrum.
(4) The main frequency amplitudes of the discrete-edge face end mill in the *X* and *Y* directions were 23.57 and 12.48, respectively. Compared with the amplitudes of 29.61 and 16.94 of the conventional face end mill, they achieved reductions of approximately 20.4% and 26.3%, respectively.

In conclusion, the discrete cutting tools not only achieve a significant reduction in cutting force during side milling but also exhibit excellent vibration resistance through the reduction in periodic impact and the amplitude of the system response. This characteristic is of great significance for improving the surface quality of processed workpieces and extending the service life of cutting tools.

### 4.3. Comparison of Processing Surface Quality

**Figure 10:**
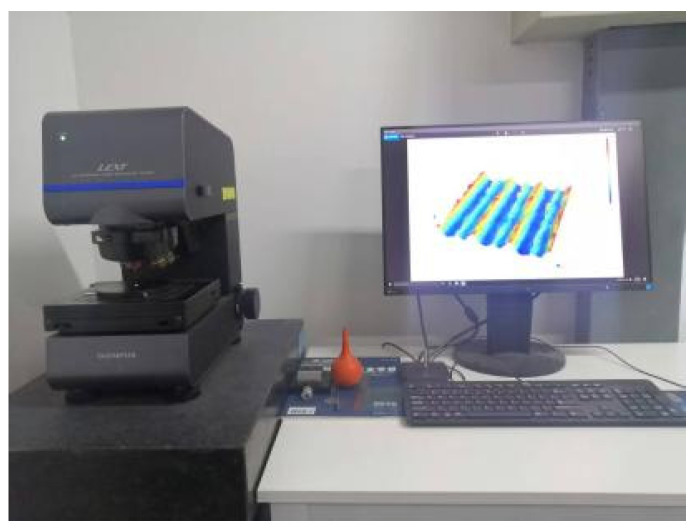
Laser scanning confocal microscope.

The three-dimensional surface morphology of 45 steel after processing was precisely measured using a laser confocal microscope (OLS5000, Olympus, Tokyo, Japan). The measurement site is shown in Figure 10. The influence of different end mills on the surface quality of the workpiece during processing was evaluated. High-precision three-dimensional information of the workpiece surface was obtained through non-contact scanning technology. After the measurement, the data were processed using OLS5000 software (Olympus OLS5100) according to the ISO 25178 standard [27], and then, the surface roughness parameters (including Sa, Sq, Sz, etc.) were extracted to analyze the differences in the microscopic morphology and quality of the surfaces processed by the two different tools.

**Figure 11:**
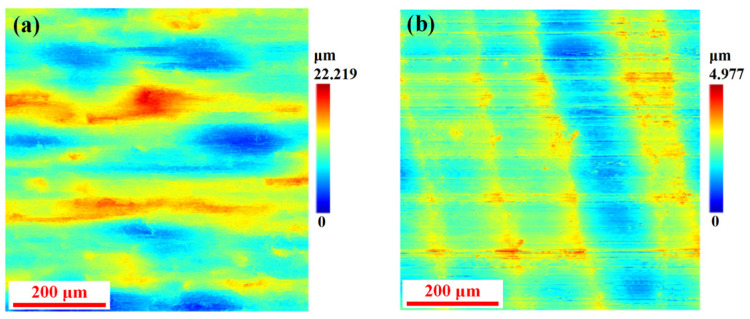
Comparison of surface morphologies processed by different tools. (**a**) The machining profile of a conventional end mill. (**b**) The machining profile of the discrete-edge end mill.

Figure 11 compares the surface morphology of 45 steel machined using a conventional end mill and a discrete-edge end mill. Under identical machining parameters, significant differences in surface morphology are observed between the two tools. The conventional end mill produces a surface with random ripples, high roughness, and poor uniformity, whereas the discrete-edge end mill yields regular periodic ripples, lower roughness, and improved surface integrity. The morphological comparison demonstrates that the discrete-edge end mill design effectively mitigates cutting load and vibration, enhances machining stability, and significantly improves surface quality and part performance.

Figure 11 shows the comparison of the surface morphology of the workpiece after processing with a traditional continuous-edge end mill and a discrete-edge end mill on 45 steel. Figure 11a indicates that the surface processed by the traditional tool has random and large-amplitude ripples, with roughness and poor uniformity; Figure 11b demonstrates that the surface processed by the discrete-edge tool presents obvious periodic ripples, with significantly reduced roughness and improved surface integrity and uniformity. This comparison directly reflects the significant advantages of the discrete-edge structure in suppressing cutting vibrations, improving processing stability, and enhancing surface quality.

To quantitatively evaluate the influence of conventional end mills and discrete-edge end mills on surface quality during the processing of 45 steel, the surface three-dimensional roughness parameters were selected for comparative analysis. The specific parameters and results are shown in Table 7.

The comparative analysis of the 45 steel surface roughness under identical cutting conditions demonstrates that the discrete-edge end mill outperforms conventional tools, reducing overall roughness (Sq decreased from 2.801 µm to 0.466 µm; Sa from 2.247 µm to 0.377 µm), lowering the peak-to-valley height (Sz from 18.903 µm to 4.164 µm), and decreasing surface complexity (Sdr from 14.347% to 1.926%). These improvements confirm that the discrete-edge structure effectively suppresses machining vibrations, enhances process stability, and improves surface quality.

## 5. Conclusions

The research systematically studied the cutting mechanism and machining performance of a discrete-edge end mill through theoretical modeling and experimental verification, leading to the following conclusions:

(1) By introducing the chip-breaking slot effectiveness function and the rotational effectiveness function, combined with the incremental cutting force superposition model, we constructed a milling force prediction model for discrete-edge cutting tools. Experiments showed that the model achieved an average cutting force prediction error of 4.82%, with a maximum error below 10%. Additionally, the errors in the main frequency position and amplitude were below 0.5% and 20%, respectively, which proved the validity and reliability of the model.
(2) The geometric design of the chip-breaking groove establishes a unique dynamic chip-breaking cutting mode, creating a synergistic mechanism of periodic segmented cutting and load dispersion. Machining performance tests showed that the discrete-edge face end mill reduces cutting forces by 7.2% and 3.2% under identical conditions. Additionally, the periodic opening and closing effect of the chip-breaking groove significantly decreases cutting force fluctuations per tooth. Specifically, the cutting force fluctuation coefficients in the XY direction were 13.5% and 21.9% lower than those of the conventional face end mill, leading to a substantial improvement in dynamic process stability.
(3) The geometric structure of the chip-breaking groove significantly improved the surface quality of the machined products. Experimental results on machining performance revealed that the discrete-edge face end mill reduced the overall surface roughness to about one-sixth that of the standard cutter. This enhancement not only boosts post-processing surface accuracy but also ensures higher stability in surface quality. The design effectively suppresses the depth and fluctuation amplitude of periodic tool marks, thereby improving the micro-forming quality of the surface.

## Figures and Tables

**Table 1:** Discrete parameter table for discrete-edge end mills.

Serial Number of the Tool	Axial Width of the Slot (mm)	Axial Spacing of the Slot (mm)	Discrete Interval Ratio of the Slot	Number of Chip Deflection Grooves on the Cutting Tool
DFC1	0.9	4.5	0.2	6
DFC2	0.9	3	0.3	10
DFC3	0.9	2.25	0.4	12
DFC4	1.2	6	0.2	5
DFC5	1.2	4	0.3	7
DFC6	1.2	3	0.4	10

**Table 2:** Cutting force identification experimental process parameters.

Machining Parameter	Experiment Value
Cutting speed, Vc (m/min)	80
Cutting depth, ap (mm)	12
Cutting width, ae (mm)	1
Each cutting feed rate during rotation, f (mm/r)	0.08

**Table 3:** Comparison of experimental values and theoretical values of milling force model for discrete-edge face end mill.

Serial Number	*F_x_*			*F_y_*		
	Experimental Value (N)	Theoretical Value (N)	Error Value (%)	Experimental Value (N)	Theoretical Value (N)	Error Value (%)
1	148.91	153.56	3.03	95.28	92.52	2.98
2	157.15	147.75	6.36	89.36	93.5	4.43
3	168.2	159.41	5.51	112.1	108.25	3.56
4	163.28	161.64	1.01	97.61	101.92	4.23
5	142.4	153.2	7.05	109.37	101.8	7.44
6	135.44	145.57	6.96	92.5	95.61	3.25

**Table 4:** Comparison data summary table.

Project	*X*			*Y*		
	Experiment	Theory	Error	Experiment	Theory	Error
Average force	146.91	154.54	4.94	99.83	93.07	7.26
Basic frequency	141.4	141.9	0.35	141.4	141.9	0.35
Main frequency amplitude	124.6	102.51	21.55	83.09	95.33	12.84

**Table 5:** Cutting experiment parameter table.

Type of Cutting Tool	Diameter (mm)	Number of Gear Teeth	Cutting Speed (m/min)	Feed Rate per Revolution (mm/r)	Side Milling Width (mm)	Side Milling Depth (mm)
Conventional end mill/Discrete-edge end mill	12	4	40	0.04	0.8	12

**Table 6:** Comparison of cutting force statistical characteristics.

End Mill	Average Force (N)		Coefficient of Fluctuation		Basic Frequency (Hz)		Main Frequency Amplitude	
	*X* Direction	*Y* Direction	*X* Direction	*Y* Direction	*X* Direction	*Y* Direction	*X* Direction	*Y* Direction
Conventional	92.85	55.53	0.74	0.73	71	71	29.61	16.94
Discrete	86.18	53.74	0.64	0.57	71	71	23.57	12.48

**Table 7:** Comparison of surface roughness parameters between discrete-edge end mills and conventional end mills.

Parameter	Discrete-Edge End Mill	Conventional End Mill	Contrastive Analysis
Root mean square height, Sq (µm)	0.466	2.801	The surface waviness of the discrete-edge end mill has been significantly reduced, being only about 1/6 of that of the standard tool. The roughness level has been greatly decreased, and the surface has become smoother.
Maximum peak height, Sp (µm)	2.152	10.093	The discrete-edge end mill significantly reduces the height of surface protrusions, indicating a significantly smaller peak value and improved surface quality.
Maximum valley depth, Sv (µm)	2.011	8.809	After processing with the discrete-edge cutting tool, the valleys become significantly smaller, the surface depressions become shallower, and deep surface defects are avoided, which is beneficial for extending the service life of the parts.
The greatest height difference, Sz (µm)	4.164	18.903	The maximum height fluctuation after processing with the discrete-edge end mill has been significantly reduced, the surface stability has been greatly enhanced, and the surface integrity has been noticeably improved.
Arithmetic mean height, Sa (µm)	0.377	2.247	The overall surface roughness of the discrete-edge end mill is about 1/6 of that of the standard cutter, greatly improving the surface accuracy after machining.
Developed area ratio, Sdr (%)	1.926	14.347	The texture complexity of the surface of the discrete-edge end mill is significantly reduced, and the surface is smoother, which is beneficial for the wear resistance and fatigue resistance of functional parts.

## Data Availability

The original contributions presented in the study are included in the article, further inquiries can be directed to the corresponding author.
